# “Just Caring”: Can We Afford the Ethical and Economic Costs of Circumventing Cancer Drug Resistance? 

**DOI:** 10.3390/jpm3030124

**Published:** 2013-07-16

**Authors:** Leonard M. Fleck

**Affiliations:** Center for Ethics and Humanities in the Life Sciences, 965 Fee Road, Michigan State University, East Lansing, MI 48824, USA; E-Mail: fleck@msu.edu; Tel.: +1-517-355-7552; Fax: +1-517-353-3289

**Keywords:** personalized medicine, targeted therapies, oncology, cancer, health care justice, rational democratic deliberation, just caring problem, health care rationing

## Abstract

Personalized medicine has been presented in public and professional contexts in excessively optimistic tones. In the area of cancer what has become clear is the extraordinary heterogeneity and resilience of tumors in the face of numerous targeted therapies. This is the problem of cancer drug resistance. I summarize this problem in the first part of this essay. I then place this problem in the context of the larger political economic problem of escalating health care costs in both the EU and the US. In turn, that needs to be placed within an ethical context: How should we fairly distribute access to needed health care for an enormous range of health care needs when we have only limited resources (money) to meet virtually unlimited health care needs (cancer and everything else)? This is the problem of health care rationing. It is inescapable as a moral problem and requires a just resolution. Ultimately that resolution must be forged through a process of rational democratic deliberation.

## 1. Introduction

It is reasonable to say that the age of personalized medicine and targeted therapies started with imatinib (Gleevec) for the treatment of GIST (Gastro Intestinal Stromal Tumor). The success of that drug was so astounding, and its perceived promise so enormous, that it made the cover of TIME magazine early in the new millennium [1]. However, this may have created an overly simplified and overly optimistic picture of how other targeted therapies could be developed for other cancers. What has become clearer over the past few years is the extraordinary heterogeneity and resilience of tumors in the face of dozens of new targeted cancer therapies. This is the problem of cancer drug resistance. 

In the next part of this essay I will summarize the research that depicts the complexity of the problem of cancer drug resistance. This needs to be placed in the economic context of the problem of rapidly escalating health care costs both in the US and in Europe, the third part of this essay. In turn that needs to be placed within an ethical context: How should we fairly distribute access to needed health care for an enormous range of health care needs when we have only limited resources (money) to meet virtually unlimited health care needs (cancer and everything else)? This is what I have referred to in my own work as the “Just Caring” problem [2]. Multiple issues of health care justice will be identified and analyzed in the last part of this essay. 

A number of researchers have suggested that the ultimate strategy for addressing the heterogeneity of cancer will be a “rational combinatorial approach” [3,4,5,6,7]. In other words, multiple drugs would be given at once to initially contain the cancer, and then they would be sequentially altered in response to the evolution of the tumor(s). This is a version of the strategy that works with HIV. But HIV is a virus, a much simpler entity to manage (as actual clinical results have proven). It requires a considerable degree of optimism to believe that this same sort of success is achievable with regard to the enormously more complex biology of cancer. But even if this optimism proves to be well-founded, the economic and ethical costs that would be incurred ought to give us pause. 

## 2. Heterogeneity, Targeted Therapies, and Drug Resistance

The heterogeneity of individual cancers has been known for several decades [8,9]. Initially that heterogeneity was identified at the histological level. Today that heterogeneity is recognized at the molecular and genetic levels [10,11]. The heterogeneity is extraordinarily complex. It is not just a matter of genetic heterogeneity of tumors among individuals, each of whom has what would have been identified in the past as the “same tumor.” And it is more than genetic heterogeneity among tumors within the same individual. Rather, the genetic heterogeneity often exists within individual tumors [10,12]. Instead of tumor growth being linear, it appears more often as branched evolutionary growth [10]. Gerlinger *et al.* performed exome sequencing on a number of samples of primary renal carcinomas and associated metastatic sites, and found that 63% to 69% of all somatic mutations were not detectable across every tumor region [10]. The major practical conclusion of this research was that (p. 883) “intratumor heterogeneity can lead to underestimation of the tumor genomics landscape portrayed from single tumor-biopsy samples and may present major challenges to personalized-medicine and biomarker development” [10]. To be more precise, the major challenge for personalized medicine is that it targets the primary driver of a tumor, and will often successfully defeat or contain that driver. But that only creates an opportunity for genetic sub-populations in a tumor to become the drivers of renewed tumor growth, somewhat in Darwinian fashion. This will usually mean that the targeted therapy is no longer effective; tumors then are described as being resistant.

The phenomenon of cancer resistance can be characterized in a variety of ways. In some instances cancers are resistant to first-line traditional chemotherapeutic agents. In other cases resistance develops in response to attempted therapies. Efforts to understand this resistance generated the research that has sought to characterize tumors in genetic terms and to identify specific biological pathways connected to specific cancers that were necessary to generate or sustain those tumors. These efforts have been aided by the development of massive parallel sequencing (MPS) capacities that have made possible the sequencing of cancer genomes quickly enough to be clinically useful and cheaply enough to be affordable (roughly $5,000). Sequencing, in turn, allows the identification of druggable targets and the “rational” development of personalized or targeted or precision medicine. Of course, what every cancer researcher knows today is that this is a very oversimplified picture of cancer therapy. 

As noted earlier, the heterogeneity of many cancers means there is most often no one target, which, if hit precisely, will result in the defeat of that cancer. Garraway and Jaenne conclude (p. 214), “The challenge of tumor drug resistance therefore represents a pervasive barrier that confounds the ultimate goal of cure or long-term control of metastatic cancer” [3]. They go on to identify the main categories of acquired drug resistance categories. These include: (1) secondary genetic alteration in a drug target; (2) a bypass mechanism, such as the activation of a parallel signaling pathway; (3) alterations in upstream or downstream effectors; (4) a pathway-independent resistance process. At present two broad strategies might be employed to address the problem of resistance. Several drugs might be used in sequence, or several drugs might be used in combination with one another. For many GIST patients imatinib proved very effective in preventing disease progression for long periods of time. But eventually it failed and other tyrosine kinase inhibitors, such as dasatinib or nilotinib, were employed in succession to slow disease progression, usually for briefer intervals. However, as Garraway and Jaenne note (p. 223), if resistance results from activation of a parallel signaling pathway, then a combination of two or more drugs will be necessary to slow disease progression [3]. As noted earlier, this is the same strategy that has successfully contained the AIDS virus for more than a decade in hundreds of thousands of individuals [4]. However, in the case of cancer this combinatorial strategy generates some potentially significant clinical problems. 

First, there are the risks of enhanced toxicity associated with various drug combinations. The monoclonal antibodies have excellent specificity in their targeting. However, this is much less true with small-molecule therapeutics that (p. 689) “commonly exhibit off-target as well as on-target effects” [4]. Also, the additive effects of several off-target drugs could potentially be very medically problematic. Minimizing that risk of toxicity might mean minimizing effectiveness as well. Second (p. 224), “the theoretical number of therapeutic combinations is vast” [3]. To be more precise, Al-Lazikani *et al.* write (p. 681): “If we consider the set of ~250 approved cancer drugs, there are 31,125 possible two-way combinations and 2,573,000 three-way combinations. For the estimated 1,200 cancer drugs currently in development the respective numbers rise to 719,400 and 287,280,400” [4]. The authors quickly add that not all of these mathematically possible combinations would make medical sense, but the numbers are still daunting, especially if we try to imagine doing all the clinical trials necessary to secure a strong evidential base. The hope for the future is that much simpler and less expensive clinical trials may be possible if researchers can develop analytically validated biomarkers for patient selection and can successfully complete the pharmacokinetic-pharmacodynamic research for identifying safe and effective combinations of these drugs [13,14].

This is only the beginning of the complexity that would have to be managed at the clinical level. Thus, Castano *et al.* write (p. 462): “The tumor ‘onco-genotype’, which defines the collection of disease-related mutations and evolves over time due to inherent genomic instability, differs among patients so that nearly every tumor cell population is unique, thus adding to the clinical challenges” [15]. What that genomic instability means in clinical practice is that there must be longitudinal assessment of the tumor state. This is necessary so that targeted therapies can be adjusted (p. 689) “as the tumor reacts dynamically to the perturbation and evolves under the selective pressure of treatment” [4]. Longitudinal assessment means that repeated tumor biopsies would have to be done. If the context is a metastatic disease process, then multiple tumors would have to be biopsied to assess how heterogeneous the target population might be and what form combinatorial therapy ought to take. The hope for the future is that non-invasive methods might be found for eliciting this information, such as assessing circulating tumor cells or other blood-borne markers, such as tumor DNA [4].

Perhaps the area of greatest deficiency so far as cancer research is concerned would pertain to biomarkers. *Predictive* biomarkers are needed to identify patient sub-groups most likely to benefit from specific targeted therapies (sequential or combinatorial). *Response* biomarkers are needed to judge in a more timely and refined way the effectiveness of specific targeted therapies, as opposed to relying upon a relatively crude indicator such as disease progression. Also important is the need to identify the most therapeutically relevant oncogenic mutations in specific clinical circumstances, *i.e.*, those most likely to be actionable or druggable. As Prasasya *et al.* note (p. 200), “Given the large number of possible mutations, developing drugs against each oncogene seems impractical; additionally, some mutations appear to be of little consequence, while others play a key role in driving a tumor’s development” [16]. To add still more to the therapeutic challenges, genetic and epigenetic features of an individual will also need to be taken into account in making suitable therapeutic judgments pertinent to specific cancers [17]. Steensma points out in this regard that “the presence of DNMT3A, NPM1, or MLL mutations influences dose response to daunorubicin, which is used to treat AML (Acute Myelogenous Leukemia)” [18]. 

## 3. The Future of Cancer Therapy: The Economic Context

No reputable researcher today believes that cancer will be defeated by some drug that will function as the proverbial “magic bullet.” Despite all the talk of targeted therapies and precision medicine, the biological heterogeneity of most cancers along with their genomic instability and the evolution of complex forms of resistance mean that medical researchers are faced with enormous challenges in trying to devise effective therapies, especially in a metastatic context. More precisely, the most that can be reasonably hoped for as an outcome of these efforts might be some significant prolongation of a reasonable quality of life with metastatic disease [19]. The hope of cure may only be a distant mirage, useful only for sustaining a research effort that generates some degree of clinical benefit. 

But there is another dimension to the challenges of cancer research that also represents an enormous challenge but that most researchers seem to ignore or dismiss. This is the economic dimension. In the very substantial review article by Lazikani *et al.* only a single brief sentence near the conclusion speaks to the economic dimension of cancer research and therapy. They write: “Finally the cost of drug combinations must also be considered in health systems that are increasingly financially constrained” [4]. 

The relevant economic context for this discussion can be quickly sketched. In the US total health spending in 2012 was about $2.8 trillion, or 17.8% of our GDP [20]. That can be compared to 1960 when total health spending in the US was only $26 billion, or 5.2% of GDP. That steep increase in GDP devoted to health care is what is of greatest concern to health policy analysts and policymakers generally. Over that fifty-year period of time average annual increases in health care costs have been roughly two and a half times the core rate of inflation in the economy. Numerous factors explain those increases in health care costs, but the dominant factor would be advances in medical technologies. As Daniel Callahan has argued, new medical technologies effectively create new medical *needs* (to be distinguished in moral terms from mere medical *wants*) [21]. If we were to ask in 1970 how many patients “needed’ bypass surgery, the correct answer would be that no one needed bypass surgery then because bypass surgery had barely been invented at the time. But in 2012 in the US we did more than 400,000 coronary bypass surgeries at a cost of $124,000 each ($50 billion), and we did an additional 1.3 million coronary angioplasties at $60,000 each ($78 billion) [22]. We also installed 120,000 implantable cardiac defibrillators at $134,000 each to reduce the risk of sudden death from an arrhythmic event [22]. There are 5.5 million individuals in the US at present in various stages of heart failure, and 550,000 new cases diagnosed each year. Patients in end-stage heart failure now have as a life-prolonging option the left ventricular assist device (LVAD) at a cost of $200,000. It is estimated that we could implant 200,000 of these devices annually. We also have in clinical testing a totally implantable artificial heart. It is estimated that we could install 350,000 of these devices annually when clinically available at a cost of $300,000 each. This is all in one area of medicine, not to mention all the new cardiology drugs or other cardiac procedures. This same story will be replicated in virtually every other area of medicine, including oncology. 

In oncology policy attention has been focused on these extraordinarily expensive targeted therapies. There are more than sixty of these drugs now with course of treatment prices ranging from $50,000 to more than $130,000. About 580,000 Americans die of cancer annually and 1.66 million are newly diagnosed annually [23]. In 2012 there were 13.7 million Americans identified as “cancer survivors” [23]. More than 3 million Americans will be actively treated for their cancer in any given year. In 2010 the total cost of medical care for cancer patients in the US was about $125 billion [23]. Assuming a 5% annual increase in costs for cancer care in the US (which is very conservative, depending upon what assumptions are made regarding access to these targeted therapies), those costs will rise to $207 billion in 2020 (constant 2010$). In that year it is projected there would be 18 million American “cancer survivors.” In 2020 projected aggregate health costs in the US are placed at $4.5 trillion, roughly 20% of anticipated GDP [20].

Noteworthy is the fact that cancer is most often a disease of the elderly, those over age 65. About two-thirds of cancer patients are over age 65. In the US this is especially significant because these are costs that are largely borne by the Medicare program, mostly funded by taxes. In 2012 the Medicare program spent about $600 billion for 47 million covered lives [20]. This program is projected to cost the federal government $1 trillion in 2020 [20], and $8.5 trillion over the ten-year period from 2013 to 2022. Driving these costs upward are the technological developments discussed above and the aging out of the post WW II “baby boom” generation, expected to swell the Medicare population to 80 million by 2030. Again, this aging out of the population implies dramatic increases in the incidence of cancer and equally dramatic increases in the cost of treating those patients, especially if these targeted therapies become very widely disseminated. Younger cancer patients will often struggle with paying for needed cancer treatments due to being uninsured or underinsured, and consequently, will often be denied access to these treatments by hospitals and physicians. But Medicare is often described as national health insurance for the elderly. It is an entitlement program that assures access to these treatments for the vast majority of Medicare patients with cancer.

Though all the statistics presented thus far are related to the US, comparable statistics can be generated for the European Union. The nations of Europe are generally spending smaller fractions of their GDP on health care, mostly in the range of 8% to 12%. Still, the problem of health care cost control is judged to be as socially and politically problematic in the European Union as in the US. The EU is faced with an aging population comparable to the US. Costly new medical technologies, including all these targeted cancer drugs, are as available in the EU as in the US. Noteworthy is that both in the EU and the US these two phenomena (an aging population and expanding life-prolonging medical technologies) interact synergistically to make the cost problem even more irresolvable. That is, greater numbers of individuals are living longer with a greater burden of chronic illness for which more and more can be done to prolong the trajectory that results in death. Marked success (nothing curative) in treating many forms of heart disease has made possible a rising incidence of cancer among the elderly as well as a rising incidence of Alzheimer’s disease (along with many other chronic degenerative disorders). One policy analyst summarized this situation accurately by saying that we are “doing better and feeling worse” [24].

What would make us “feel worse” by the often trumpeted successes associated with the development and dissemination of these targeted, personalized cancer treatments? The short answer is that in the vast majority of cases these drugs yield very marginal benefits at a very high cost [25]. For many of these $100,000 drugs median gains in survival are measurable in weeks or months [26,27,28]. Fojo and Grady, for example, call attention to cetuximab in connection with non-small cell lung cancer [29]. The median gain there is six weeks for $100,000. In cost-effectiveness terms, that means we are willing to spend $800,000 to gain an extra year of life [29]. Economists would point out that this could hardly be a reasonable or prudent use of social resources, especially if numerous other life-years could be purchased at a tiny fraction of that cost by allocating those dollars to meet other life-prolonging health care needs. The cost of saving a life-year for an HIV-positive patient with a four-drug combination would be about $30,000. Why would an economically rational society not make these more reasonable re-allocations of health care resources?

Several brief answers might be given to this last question. First, these targeted cancer therapies are being given to patients faced with what will likely be a terminal outcome. They have no other options that are likely to be effective in prolonging their lives. These therapies are regarded as “last chance” therapies to which greater social value is attached than other kinds of economic goods [30,31]. Second, it is sometimes vocalized and more often silently affirmed that in our society human life is “priceless”. The intent behind this affirmation is that it is unseemly to make an *explicit* social decision to deny someone a life-prolonging therapy merely because it cost too much money [32]. The reader will note that “explicit” is italicized because in the US (to what should be our great shame) we are quite tolerant of less visible implicit ways of denying individuals access to expensive life-prolonging care. We ration by ability to pay. If individuals lack the financial resources to pay for such care, then we “respect their autonomous choice” to deny themselves such care. Then it is *their* choice, not a social choice that is imposed upon them by legislative or administrative fiat. Third, cancer is greatly feared as a disease. One in three Americans will receive a diagnosis of cancer sometime in the course of their life. That creates substantial social and psychological pressure to make certain that cancer research and cancer therapies are well funded, even if that funding does not represent the most prudent use of social resources. 

We noted above a kind of social urgency, perhaps rooted in social compassion that is attached to “last chance” therapies. Perhaps it is not really unreasonable for a society as wealthy as our own to pay $100,000 for patients who need access to these targeted therapies. However, our discussion of drug resistance in the face of the heterogeneity and genomic instability of many cancers, and the emerging commitment among researchers to follow the AIDS paradigm in attacking cancer, means that we ought to embrace the combinatorial strategy [33]. This will raise something of a conundrum. How will we know what is *really* the “last chance” therapy that deserves a very generous dose of social compassion? If we have in mind the sequential combinatorial version of targeted therapy, then we would be administering one of these very expensive targeted therapies until it was clear that the cancer was progressing, at which time we would switch to another of these drugs until the cancer progressed again, and perhaps there could be three or more such efforts before a patient succumbed. But then we are talking about expenditures of several hundred thousand dollars, each of which might be yielding only a marginal benefit for that patient. 

The same will be true, perhaps at even greater expense, if three targeted therapies are administered simultaneously, as with HIV triple therapy, in an effort to defeat multiple drivers of a cancer (hoping for longer periods of progression-free survival). Still, as with HIV therapy, the first combination will most likely be defeated by the cancer and require a different combination of these targeted drugs, now aimed at the emergent drivers of the cancer. As that combination is defeated yet another combination can be tried. Two possible concluding scenarios might be imagined at this point. In the first scenario the patient succumbs after cancer variants emerge for which there are no more targeted therapies. In the second scenario the cancer is kept at bay as a chronic disease with constant infusions of combinations of these targeted drugs for some number of years. In this latter scenario we might have to refrain from describing the overall outcome as a marginal benefit, but the cost of achieving that outcome might be more than a million dollars per person. When considered in aggregate terms the costs become economically staggering. 

We might try to imagine the situation this way. There are almost 600,000 patients in the US who die of cancer each year. If that last year of life cost $100,000 for one or more of these targetable drugs, that would represent an expenditure for that cohort alone (no other cancer care for any other cancer patients) of $60 billion. But if we were successful in giving all those individuals an extra year of life for another $100,000 expenditure, that would raise the annual cost of providing cancer care to these terminally ill patients to $120 billion. If we achieved modest five-year success with this combinatorial strategy (“modest” relative to the fifteen year gains of many HIV patients on triple therapy), and if each of those extra years required “only” $100,000 worth of these drugs, then in year five we would be sustaining three million cancer patients at a cost of $300 billion per year *only for addressing their cancer needs*. If these patients had other health care needs, some of which might be related to side effects of prolonged use of these target therapies, then that would add to the health costs of caring for these patients. Would the social justification for such massive expenditures be accurately justified by appeal to the notion of “last chance” therapies? Or, to ask our question another way, would a just and caring society with limited resources to meet virtually unlimited heterogeneous health care needs be morally obligated to provide social funding for all these target therapies for these cancer patients? This is the most fundamental moral issue that must be addressed. 

## 4. The Future of Cancer Therapy: The Ethical Challenges

The problem we are addressing is what is usually referred to as the problem of health care justice. If all health care needs in our society cannot be met, then how can we fairly decide which needs ought, morally speaking, to be met and which can be allowed to be unmet, at least so far as social resources are concerned? This is the problem of health care rationing, sometimes referred to as the priority-setting problem. I have argued, as have many other medical ethicists and health policy analysts, that the need for health care rationing and priority-setting is inescapable [2,34,35,36,37]. As long as medical technology continues to advance and to create new health needs, we will never have sufficient resources to meet all the health needs of our society. So choices will have to be made; priorities will have to be set. How can those choices be made fairly and justly?

### 4.1. Conceptions of Health Care Justice

Those who are on the libertarian side of the political spectrum will argue that rationing is inherently unjust, if by “rationing” we mean that some governmental body is going to deny individuals access to needed health care. From their perspective, if individuals can afford very expensive life-prolonging health care, then that is what gives them a just claim (or liberty right) to that resource, even if they are paying $100,000 for a few extra weeks of life and the rest of society regards that as a very foolish expenditure. Of course, as things are now, the Medicare and Medicaid programs in the US represent social resources generated through a tax mechanism. For libertarians that in itself is completely unjust because taxes are coercively extracted from citizens [38]. Charitable organizations created to meet health care needs of various sorts are perfectly respectable because resources are freely given to such organizations. 

Critics of libertarians will respond that libertarians are being disingenuous, that what they are really endorsing is rationing by ability to pay. Part of what is morally objectionable about relying upon ability to pay to determine access to health care is that our health care system is largely a product of huge social investments by everyone who pays taxes in medical research, medical education, and construction of our major health care facilities. It would be unjust to deny access to lower-paid workers to these socially generated resources while more fortunate individuals had privileged access. Moreover, though libertarians decry with the hyperbolic rhetoric of “death panels” the health reform and health care cost control efforts of the Obama Administration, the fact of the matter is that in the US 24,000–42,000 excess deaths annually are linked to individuals being uninsured or underinsured [39]. That is, these are individuals who would be alive today but for the fact that they could not pay for the life-prolonging care that they otherwise needed. Though consequences such as this are morally problematic, the libertarian perspective has some morally reasonable elements. More specifically, it may be the case that $100,000 targeted cancer drugs yielding gains in life expectancy measurable in weeks or months ought not to be socially funded as a requirement of justice but that ability to pay should determine access to that resource.

There are several other prominent conceptions of health care justice that require brief discussion. Each of them, I would argue, is morally reasonable in some respects but not morally reasonable if seen as the exclusive arbiter of just approaches to health care rationing or priority-setting. Thus, the utilitarian perspective on health care justice says that we ought to spend our limited resources in ways that will yield the greatest health good for the greatest number. If many of these targeted cancer therapies yield only marginal gains in length of life or quality of life, then that is neither a wise nor just use of limited social resources from a utilitarian perspective [40]. Unfortunately, health care needs are not distributed uniformly across the population or across the lifespan of individuals. Some individuals, such as patients with more serious forms of cystic fibrosis, will require a million dollars’ worth of health care just to reach age twenty when they will die. Many egalitarians will want to argue that we are morally obligated, as a matter of justice and equal respect for all individuals, to meet the health care needs of those patients so long as we have effective medical technologies for them [41]. As long as these therapeutic technologies are effective, cost is morally irrelevant as well as the quality of life of these individuals and any commitment to maximize social welfare. That is, this example shows one respect in which the utilitarian perspective is morally inadequate.

The defining feature of the egalitarian perspective on health care justice is equal concern and respect for all. However, that abstract commitment generates a variety of versions of egalitarianism with very different practical outcomes. There is what is called *strict* egalitarianism. This is the view that whatever any one individual receives in access to needed health care is what every other individual with that same condition has a just claim to [41]. Again, there is an intuitive rightness and fairness about this abstract principle. But the critical question can be raised: Does that mean that an individual with advanced Alzheimer’s and late-stage heart failure would have as much a just claim to a $200,000 left ventricular assist device as a 55-year old individual with advanced heart failure and otherwise good health? An affirmative answer to this question would seem morally unreasonable, given enormous other unmet health care needs in society. Equally unreasonable, many would argue, would be denying this life-prolonging technology to the 55-year old. Still, John Harris, a strict egalitarian writes (p. 101): “Each person’s desire to stay alive should be regarded as of the same importance and deserving the same respect as that of anyone else, irrespective of the quality of their life or its expected duration” [42]. This has obvious relevance to how access to these very expensive targeted therapies ought to be determined.

There are *moderate* forms of egalitarianism. But the critical question remains: What should be equal? Should resources [43] or outcomes [44] or opportunity [45] be equalized? Each view has different practical implications so far as access to needed health care is concerned. Should everyone be given guaranteed access to one million dollars of health care for a lifetime? This could be hugely extravagant for some and completely inadequate for others, all depending upon the health care problems individuals are afflicted with, either as a result of nature or accident or bad choices. Some children might require a million dollars in cancer care for childhood leukemia, only to be faced with another defeasible cancer in their thirties. Are they then justly denied that needed care if they are unable to come up with another $200,000 from their own resources? 

Should everyone be assured access to whatever effective health care is available, no matter what the cost, if it is needed to achieve a normal life expectancy? That would address the problem in the prior paragraph. We could somewhat arbitrarily pick age seventy-five as that number. This view has the moral advantage of being sensitive to a large range of unfortunate life circumstances. But it would seem to require huge social expenditures for very marginal gain for individuals below age seventy-five. Individuals might receive several targeted cancer therapies in succession for a net gain of only an extra year of life at a cost of $300,000. Again, if there are other large unmet health care needs in society, expenditures such as that seem neither just nor prudent. In addition, we would be left with the unresolved question of what the just claims to needed health care would be for individuals beyond age seventy-five. Would that be left entirely up to individual ability to pay? That would look like ageism, unjust discrimination against the elderly. 

Norman Daniels argues for a fair equality of opportunity account of health care justice. His view is that individuals have a just claim to whatever health care would restore them to the “normal opportunity range” of their society [36]. This view has an intuitive plausibility. It says, for example, that we owe little in the way of life-sustaining care to patients who are in a persistent vegetative state, or the late stages of a dementing process, or the late stages of a terminal illness because the normal opportunities of life are no longer available to them, at least in terms of current medical capacities. They are owed comfort care. To some extent this view establishes just limits to needed health care. But it may not do enough to control access to very costly marginally beneficial care outside these three sets of circumstances.

### 4.2. Personalized Cancer Therapies: The Challenges of Justice

The above views delineate the broad framework of the problem of health care justice. We now turn to some of the justice-related ethical challenges specific to personalized cancer therapies. We will focus our attention on the combinatorial strategy because the current medical argument sees such a strategy as being necessary, given the heterogeneity problem. Someone might argue that if HIV+ patients have a just claim to triple and quadruple drug regimens that stretch out for two decades, then cancer patients ought to have a just claim to multiple combinations of targeted therapies aimed at controlling the cancer. However, there are some major morally relevant differences. The cost of HIV drug combinations is about $30,000 per year while the cost of many of these targeted therapies exceeds $100,000 for a single drug that yields only several months of gain in life expectancy [46,47,48]. This implies a cost-effectiveness number in many cases of several hundred thousand dollars for an extra year of life. It would not be obvious that cost-effectiveness numbers at that level represent either a wise or a just expenditure of limited societal resources.

It is also justice-relevant that the AIDS drugs are very effective. They keep HIV at bay (and the patient reasonably healthy) for years and years. Targeted therapies are most often only very marginally effective; median gains in life expectancy relatively rarely get beyond one year. Still a defender of these targeted therapies might argue that from the point of view of aggregated social costs per patient, the AIDS regimen can cost $600,000 if individuals gain twenty extra years of life. In contrast, cancer patients on current single targeted agents might gain less than an extra year of life, but the social cost will be “only” $100,000. Then again, if we start using multiple targeted therapies in combination and in succession at a cost of $200,000 per combination per year for three years, we would have equaled the AIDS combination figure. That would suggest that these targeted therapies are much less socially productive of saved life years than current AIDS regimens. This does not yield the moral judgment that funding these targeted therapies for cancer is unjust. But it does suggest that they ought (for reasons of equity and efficiency) to receive lower priority relative to drugs used to treat HIV+ patients.

Our judgments of health care justice need to be fact-sensitive. The facts now are that these targeted cancer therapies are extraordinarily expensive, both at the individual level and at the level of aggregated social costs. If the facts changed, if the costs of these drugs decreased very substantially and if the gain in life expectancy also increased dramatically, then different health care justice judgments would be warranted. In the meantime it is noteworthy that no moral argument would justify regarding cancer and its therapeutic modalities as being somehow morally special, *i.e.*, worthy of unlimited commitments of social resources. Instead, the question that needs to be addressed is the issue of how high a priority these targeted cancer therapies ought to have relative to all the other life-prolonging technologies available in any advanced society for addressing a broad range of life-threatening medical problems.

Our judgments of health care justice need to be fact-sensitive. The facts now are that these targeted cancer therapies are extraordinarily expensive, both at the individual level and at the level of aggregated social costs. If the facts changed, if the costs of these drugs decreased very substantially and if the gain in life expectancy also increased dramatically, then different health care justice judgments would be warranted. In the meantime it is noteworthy that no moral argument would justify regarding cancer and its therapeutic modalities as being somehow morally special, *i.e.*, worthy of unlimited commitments of social resources. Instead, the question that needs to be addressed is the issue of how high a priority these targeted cancer therapies ought to have relative to all the other life-prolonging technologies available in any advanced society for addressing a broad range of life-threatening medical problems.

In the US there are other problems of health care justice associated with these targeted therapies. Different health plans may cover these drugs, but they might identify them as a Tier Four drug. This will also be true for older patients in the Medicare program who must purchase a Medicare Advantage plan for prescription drug coverage. These individuals could be faced with a copay requirement of 30% for these drugs. If a drug costs $100,000 an individual would have to pay $30,000 of those costs. The practical consequence of this arrangement is that a large portion of Americans would not be able to afford these drugs. It could be argued that this is an outcome that is not especially morally objectionable. After all, many commentators have noted that these drugs are not cost-effective and yield only marginal benefits. It is not as if these drugs will cure a metastatic disease process. If wealthier individuals wish to spend their money in this way, then those choices ought to be respected. There is no injustice there. However, that conclusion is too quick. The fact is that these wealthier individuals are paying only 30% of the cost of these targeted therapies from their own pockets; the remainder of the cost is actually being covered by all the individuals who were unable to afford the drug themselves in the form of their basic insurance premiums. To correct for what seems like an obvious injustice, these targeted therapies and other such costly marginally beneficial interventions would have to be accessible only through a separate insurance mechanism with premiums paid out of pocket by individuals who believed such benefits were worth it to them.

Most European nations do not have to address the above issue because copays are a rare phenomenon. However, Europe and the US will have to address what I have discussed elsewhere as the “ragged edge” issue. This is a term that Daniel Callahan introduced into the literature [21]. It means that there are often no bright lines “in nature” that separate health care interventions that are cost-effective from those that are not cost-effective. Consequently, when health care rationing decisions need to be made and some sort of cutoff for social funding is put in place there will be patients on the other side of that line who will wonder how it could possibly be just to deny them what they might regard as the only life-prolonging resource available for their medical problem. That sort of political and ethical awkwardness can be avoided by not drawing any lines and funding everything that does any medical good for any patient in specific medical circumstances. That, however, represents a denial of the need to control escalating health care costs. 

In the UK the National Institute for Clinical Excellence (NICE) uses roughly $50,000 per Quality-Adjusted Life-Year as a criterion for determining which new therapeutic interventions would be funded by the National Health Service [49]. In the US the present consensus would endorse a figure around $100,000 for making the same judgments, though this does not represent any “official” commitment to such a figure by either government or business. However, as the genomic revolution in medicine advances the “ragged edge” is appearing (or threatens to appear) in numerous medical circumstances. 

Schneider *et al.*, for example, looked at bevacizumab and paclitaxel as a treatment for metastatic breast cancer compared to paclitaxel alone [50]. Median survival in these two arms was virtually indistinguishable, 25.2 months *vs*. 26.7 months. It would cost $100,000 to buy those six extra weeks, which is equal to a cost per QALY of $800,000. However, 7% of the women in the bevacizumab cohort with a specific VEGF genotype achieved median survival of 50 months. They gained more than two extra years of life for a cost per QALY of only $50,000, which seems quite reasonable. Another 11% of these women with a different VEGF genotype achieved a median survival gain of 30.5 months, or five extra months relative to the paclitaxel group alone. That works out to a cost per QALY of $240,000, much less of a reasonable use of limited health care resources. All the other genotypes achieved lower gains in survival (and higher cost-effectiveness numbers). The ethical issue is this: If we have reliable predictive biomarkers for such large differences in outcomes, then is it “just enough” if we only provide public funding for a drug such as bevacizumab for those most likely to gain a sufficient prolongation of life to justify the cost of the drug? 

As things are now, *i.e*., very little predictive genetic testing related to expected outcomes and only marginal reliability, we provide these very expensive targeted therapies more or less indiscriminately to patients for whom these drugs are likely to provide some degree of clinical benefit, *i.e*., some progression free survival [51]. If predictive genetic testing were to show that a cancer patient would almost certainly derive no clinical benefit from one of these drugs, then there is clearly nothing unjust about failing to offer the drug to such patients. But should we identify, for the sake of a fairer and more prudent use of health care resources, some minimal clinical gain that would be sufficient to justify the very high costs associated with these targeted therapies? 

Here are some other examples of ragged edge issues that ultimately raise this same ethical issue. What sort of clinical benefit should count toward justifying these large costs? In one recent study of HER-2 metastatic breast cancer patients, one group was given letrozole alone while the other group was given letrozole plus lapatinib [52]. There was a five-month difference in progression free survival between the two groups, which looks like a somewhat significant clinical benefit. But overall survival showed no significant difference between the two groups. Lapatinib has an annual cost of about $48,000. Should lapatinib be provided in these clinical circumstances by a just and caring commissioning group in the UK, or a managed care plan in the US, or a sickness fund in Germany? What measure of clinical benefit is most appropriate in these circumstances? 

With regard to this last example as well as our prior example we note that what is being compared are median gains in progression free survival or overall survival. There are tails that go in both directions. Suppose, for example, that we had social agreement that one-year survival was necessary to justify a social expenditure of $100,000 for one of these targeted therapies. Suppose we also knew that 5% of the patients in the five-month median survival cluster with bevacizumab and a specific VEGF genotype did in fact survive a year. We have no way of identifying before the fact which individuals might be in that 5% group. Would considerations of health care justice require that we then provide bevacizumab to that entire cohort in order to satisfy the just claims of that 5%? Or would we be permitted to disregard that 5% and not be judged unjust? Would it be morally permissible and/or economically desirable to avoid doing the research and identifying the predictive biomarkers that would give us the knowledge that generates these sorts of painful ethics issues? Should research such as this be seen as a threat to the social value of solidarity in a European context?

Our discussion started with the problem of drug resistance, genomic instability, and heterogeneity in many tumors. The emerging strategy for addressing this problem is to use combinations of these targeted therapies with the goal of defeating several drivers of a metastatic cancer at once in order to increase both progression free survival and overall survival. The ragged edge questions that arise in this context would be the following: If the cost of such combination therapies is substantially greater than the cost of a single targeted drug, should the gain in expected overall survival be proportionately greater as well? If, for example, the cost for an initial combination were $200,000 would it be just and reasonable to expect two-year survival in order to justify social coverage of these costs? If so, should we be doing research aimed at determining and identifying whether there are sub-groups of, for example, non-small cell lung cancer patients who would meet this survival goal and others who might fall far short? If these others fall short because of the more rapid development of drug resistance and cancer progression, does health care justice really require that we then try an alternative combination of these targeted therapies, *and do the research that would inform us of the alternate combinations of drugs that are likely to achieve additional progression free survival or overall survival?* If so, how many such combinations are metastatic cancer patients entitled to as a matter of health care justice? Are they, for example, entitled to any combination, no matter how many combinations, that yield at least three additional months of predicted survival? 

The obvious problem I am calling attention to is that a strategy such as this might be able to achieve five years of survival that these patients would otherwise not have, which is a substantial gain in life expectancy. But the cost of such an achievement at current drug costs might easily exceed a million dollars per case. This is a cost that might be socially affordable for relatively small cohorts of patients. But the 580,000 cancer patients who currently die each year in the US (or the 1.3 million in the EU) would all be candidates for such life-prolonging efforts [53]. The aggregate annual costs *for just these cohorts of metastatic cancer patients* would readily exceed $100 billion per year in the US, and would threaten to “crowd out” many other health care needs with less social visibility by making such large demands on health care resources [54]. What sorts of limits and trade-offs are fair in these circumstances, all things considered?

We noted earlier that cancer is primarily a disease of the elderly. We also noted that a growing proportion of the populations of both the US and the EU are aging, and that the elderly in the US consume three times as much health care per capita as the non-elderly. Costly advances in cancer treatment would further skew that proportion to the potential disadvantage of the non-elderly. This is another problem of health care justice if we believe that all in our society ought to have a fair opportunity to achieve a normal life expectancy if that is medically possible. But then that raises an additional ragged edge question. Should we permit the non-elderly with metastatic cancer greater access to these future combinations of targeted therapies, even at a substantially greater per capita cost, simply because we ought to accord them an opportunity to achieve as close to a normal life expectancy as medically possible? 

### 4.3. Assumptions and Limitations

As noted earlier, our ethical judgments in medical ethics and health care policy are fact-sensitive. If the facts on which certain judgments of health care justice are based are substantially altered, it will be necessary to alter those judgments. One reviewer has noted that if the costs of these targeted therapies are very substantially reduced, perhaps as they go off-patent, then the problems of health care justice outlined in this essay would be diminished as well. That would be fundamentally true, assuming that those costs were reduced by 50%–70%. Again, the reader needs to keep in mind that the economic and moral concern is not cost as such but cost-effectiveness. A drug that cost only $25,000 for a course of treatment that yielded an average gain in life expectancy of only three months would still have a cost-effectiveness value of $100,000 for a gain of one year of life. Again, we can hope that future medical research will improve dramatically the medical effectiveness of future targeted cancer therapies. It may well be the case that the combinatorial approach that has worked so well in addressing the AIDS issue will yield equally dramatic results for cancer. We have to wait and see. There are no guarantees in this regard. Moreover, cancer is proving to be enormously more complicated to defeat than the AIDS virus. It was only fifteen years after the AIDS virus was identified that protease inhibitors were proven effective in containing the virus. A comparable story cannot be told with regard to cancer. After more than fifty years of intense cancer research metastatic cancer remains as deadly as ever, though we clearly have a much more sophisticated understanding of why metastatic cancer is so difficult to defeat.

This same reviewer noted triple therapy for AIDS is now available in the developing world, the implication being that the cost of these drugs has decreased so substantially that almost anyone with the relevant medical need has access to these drugs (therefore, no problem of justice). However, that comparison is misleading if the comparison is intended to suggest that this could be the future of cancer combination therapies. Considerable political effort was required to achieve those cost reductions, including all manner of special protections to prevent those AIDS drugs from being re-imported into Europe or the US. Further, many European countries have been able to successfully bargain with pharmaceutical companies for very large discounts on these AIDS drugs; I am not aware of comparable success when it comes to these targeted cancer therapies, primarily because, unlike anti-depressants, there are not multiple targeted cancer therapies in a class that can be easily substituted for one another. In the US both the AIDS drugs and targeted cancer therapies remain much more expensive than in Europe. This is because US law forbids our Medicare program from bargaining with pharmaceutical companies as an organization with 43 million covered lives. US law also forbids Medicare from refusing to accept for coverage any of these new cancer therapies on the basis of cost-effectiveness, which is an option generally available in Europe. This too is a moral problem as we look forward over the next ten years. As long as that remains true, the justice issues outlined in this essay will remain salient and serious in the US context.

Though this essay has been written mostly with US circumstances in mind, the cost of these cancer therapies is a serious moral and economic problem for much of Europe as well, even though every European nation has annual health expenditures more than six percentage points below what the US spends on health care as a fraction of GDP. Even though this essay has offered speculative moral judgments that look out over the next ten years, the justice questions are real and serious today because the costs of these targeted therapies are what they are today. To illustrate my point, I noted earlier the role of NICE in assessing new drugs for inclusion or exclusion by the British National Health Service. NICE has judged that some number of these targeted therapies yield too little good at too high a price to justify inclusion by the NHS. Consequently, the Cameron government created a 200 million pound fund to underwrite the costs of these drugs. This was an act that many described as political opportunism. We pass over that judgment. Policy analysts estimate that if all cancer patients in the UK who could potentially benefit to any degree from these drugs were to be covered, this fund would have to have 600 million pounds. Consequently, the local Strategic Health Authorities (SHA) are charged with determining which patients will or will not have access to these drugs through this special funding mechanism. Each SHA is free to determine what criteria it will use to make such judgments, which is a problem of health care justice because of the potential for arbitrary judgments. Equally ethically problematic is the fact that the Cameron government did not provide 200 million “new” pounds for that fund. Instead, at least 50 million pounds were taken from home health care funding [55]. At the very least it is reasonable to ask what the justice-based argument would be for that trade-off.

Finally, to return to the cost issue, much of the cost of these drugs is associated with their development. If researchers are successful in discovering a “truly rational” combinatorial approach to the targeted of these drugs, that could result in substantial reductions in the cost of these drugs. As I am using the phrase “truly rational” I am suggesting methods of determining the likely success of drug combinations that are extraordinarily reliable in predicting safety and effectiveness with much smaller clinical trials. Again, we simply have to wait and see whether those engaged in such research efforts can build predictive models capable of yielding that reliable medical knowledge. In the meantime the justice issues remain relevant because the cost issues are unresolved.

## 5. Conclusion: Health Care Justice and Rational Democratic Deliberation

I will not conclude that there is some simple and powerful moral principle that will address all the issues raised in this essay. There is not. On the contrary, all the conceptions of health care justice discussed above might have some reasonable relevance to the questions we have raised. However, as I have argued at length elsewhere, none of these conceptions of health care justice is capable of yielding a “just enough” or “compassionate enough” response to all the problems of health care justice related to the very complex problem of health care rationing and priority-setting [2]. All of these conceptions are too abstract to be suitably responsive to the very complex details of the problems of health care rationing in a clinical setting. To be clear, they do have moral utility, but it is limited. Among other things, it will often be unclear which of these conceptions of health care justice is most reasonable and most fitting for a particular rationing problem, such as any of the ragged edge issues raised above. Further, it is not the case that we just need to think about these issues longer and harder, bringing the right sort of expertise to bear, and then finding “the” fairest and most reasonable answer. It is likely closer to the truth to say that often there will be multiple “just enough” or “compassionate enough” responses to these rationing issues, and we collectively need to decide which response we are willing to accept as being “just enough” for our future possible selves whose specific health needs are unknown to us at the moment.

In the United States we are always tempted to “individualize” solutions to social problems in order to protect the liberty of individuals. But this is precisely what will not work if we are to have a just and feasible and affordable resolution of the problem of health care rationing. Our sense of justice is essentially a social construct. We cannot have just outcomes for a distributive social policy if everyone gets to decide on their own what they think is just for them. We have to have some form of *social* agreement. If this social agreement is going to be a fair agreement, this will require a *rational deliberative* process, as opposed to normal interest group politics where agreements are a product of relative political strength which can be used to impose an agreement on weaker social groups. Again, the problem of justice is a *moral* problem, which means this deliberative process must involve treating all with equal moral respect. In practice that means that no one is entitled to superior access to needed health care because of their social status or political connections. The rationing issues we have identified must be addressed rationally. That is, we must give to one another what the philosopher John Rawls refers to as “public reasons” to justify the specific rationing rules and protocols we would recommend [45]. The defining feature of public reasons is that they are not logically attached to a religious or ideological view that others might reasonably reject. We recognize that we live in a liberal pluralistic society, so we need to give one another reasons that any citizen in such a society could accept as reasonable. Thus, I cannot refuse to provide funding for some legitimate medical intervention as part of a national health plan because the Catholic Church is opposed to that intervention for essentially religiously rooted reasons.

What makes various health care rationing judgments presumptively just is that they are public or transparent; they are impartially created and enacted; they are freely and rationally self-imposed. A rational democratic deliberative process suitably inclusive and representative of all who would be affected by such judgments will satisfy those conditions. Let me offer a brief illustration of what ultimately must be generalized across our entire health care system. 

We start with the recognition that the vast majority of Americans are concerned about health care costs. Getting rid of “waste and inefficiency” in the health care system will not be sufficient to control escalating health care costs because emerging and desired new health care technologies are the real drivers of these escalating costs. That means we must identify marginally beneficial health care that costs much more than the benefits are worth. So we must be willing to give up such care for our future possible selves if in the future we found ourselves in clinical circumstances where such care, a three-drug combination for our cancer that cost $200,000 and yielded only five extra months of life, would otherwise be something that could be offered to us. 

Most people might actually want such care if they actually found themselves in those circumstances. But the deliberative process is occurring among citizens who are essentially healthy, who are behind what Rawls [45] would think of as a health care “veil of ignorance.” Virtually none of us know what the health problems are that we might have to face over the next thirty or forty years. We might have inklings based upon family history or even some actual genetic testing. But these are just inklings to which great uncertainty is attached. We might be especially anxious about cancer, so we might endorse paying $200,000 for that three-drug combination. We might be opposed to those who would want to deny access to those drugs at social expense. We might be less concerned about heart disease. So we might oppose a new drug for end-stage heart disease that cost $200,000 and yielded on average only five extra months of life. We might argue that was not worth it. But this is where moral and rational consistency must shape this deliberative process. Can a good reason, a morally compelling justice-relevant reason, be offered for making a distinction between these two sets of circumstances? It is not obvious that such a reason could be offered. Hence, if someone was concerned now about controlling present and future health care costs to them, they would have to reject these hyper-expensive drugs in both circumstances. 

We should make clear that the deliberative process is informed by the best medical knowledge available at a point in time. This is also part of what makes the deliberative process rational. As for ragged edge issues, as noted earlier, lots of possible choices could be made, all of which would be morally acceptable. That is, they would be roughly just because there would be no perfectly just choice that could be made. The world of health care is just too complex for that to be possible. Still, what is necessary is a *collective* social choice that reflects what the deliberative process judged to be a fair and reasonable trade-off. This is not something that can be left to the self-serving whims and rationalizations of individuals. Suppose that someone at age forty participated in these democratic deliberations and was opposed to any limitations on access to marginally beneficial cancer, but his views reflected a very minority position. He is now eighty and has a late stage cancer for which he is being denied at social expense one of these combinations of cancer drugs that will yield only five extra months of life for $200,000. Has he been treated unjustly? We could say he was unfortunate, though he has achieved age eighty. All others in similar circumstances are faced with the same denial; he is not a victim of some form of social discrimination. In the forty intervening years, in fact, thousands of individuals were denied similar drugs for similar reasons. The money saved was put into cancer research aimed at improving dramatically the effectiveness of advanced cancer therapy for some individuals who happened to have the right genotype or whose cancer proved to be especially responsive. In fact, we can imagine that this gentleman was a beneficiary of this research at age seventy. Under these circumstances he cannot reasonably claim that the deliberative process yielded an outcome that was unjust for him.

Ultimately, a well-constructed democratic deliberative will yield outcomes that are respectful enough of individual liberty while protecting the framework of justice that must hold a society together. It is also congruent with the European commitment to solidarity [56], otherwise potentially threatened by the destabilizing effects of personalized medicine and escalating health care costs.
